# Effect of *Cassia fistula *L. aqueous extract in maternal reproductive outcome, some serum indices and fetal anomaly frequency in rat

**DOI:** 10.22088/cjim.13.3.475

**Published:** 2022

**Authors:** Saeed Hakiminia, Zahra Esmaeeli, Ali Akbar Moghadamnia, Seyyed Gholam Ali Jorsaraei, Farideh Feizi, Sorayya Khafri, Zahra Memariani, Hoda Shirafkan, Seyyed Ali Mozaffarpur

**Affiliations:** 1 *Student Research Committee, Babol University of Medical Sciences, Babol, Iran*; 2 *Department of Pharmacology, Babol University of Medical Sciences, Babol, Iran*; 3 *Department of Anatomical Sciences, Babol University of Medical Sciences, Babol, Iran*; 4 *Department of Biostatistics, Faculty of Medicine, Babol University of Medical Sciences, Babol, Iran*; 5 *Department of Persian Medicine, School of Persian Medicine, Babol University of Medical Sciences, Babol, Iran *; 6 *Health Research Institute, Babol University of Medical Sciences, Babol, Iran*; 7 *Traditional Medicine and History of Medical Sciences Research Center, Health Research Institute, Babol University of Medical Sciences, Babol, Iran *

**Keywords:** Laxatives, Pregnancy, Cassia, Herbal medicine, Teratogenesis, Persian Medicine, Complementary medicine, Toxicity

## Abstract

**Background:**

*Cassia fistula* was used traditionally as laxative in pregnant women. Nevertheless, its fetal and maternal effects in pregnancy have not been studied yet.

**Methods:**

Oral (Lethal Dose, 50%) LD50 was determined in mice. In addition, a control group, pregnant rats in other 5 experimental groups (n=12) received orally *C.* *fistula *aqueous extract (500, 1000 and 2000 mg/kg), tween80 (10%) and distilled water during pregnancy up to the delivery (21-23 days). Some serum indices were evaluated in maternal blood samples after delivery. Histopathologic and histomorphometric evaluations were performed on the selected slices of newborn rats.

**Results:**

Anthraquinone‎ content of the aqueous extract was 0.34% w/w. Oral LD50 was obtained more than 5000mg/kg. No abortions and newborn anomalies were observed in groups. The height and weight of the offspring were significantly reduced by the administration of 500, and 2000 mg/kg of extract compared to control. There was no significant change in maternal blood urea and creatinine. Higher concentration (2000mg/kg) led to ALT elevation. ALS levels decreased dose-dependency in treatment groups comparing to control. Histopathological findings showed significant lung vascular congestion, and hypertrophy of heart in group tween80, and significant hepatic parenchymal inflammation in tween80 and 2000mg/kg and 1000mg/kg groups. In all tissues of all groups, malpighian body area and bowman’s capsule space significantly increased compared to the control group.

**Conclusion:**

It seems *C.* *fistula* extract is safe in pregnancy. Because of confounding role of tween80 in histopathological finding, more research is necessary.

The golden rain tree or *Cassia fistula* L., a member of the Fabaceae family is one of the plants which is traditionally used for its mild laxative property especially in children and pregnant women (1). Today different biological properties of *C. fistula* including laxative (2), antioxidant, hepatoprotective (3), imunomodulator(4), wound healing, and antifungal effects, etc., have been reported in numerous studies (5). *C. fistula* (“Foloos” in Persian) is one of the 134 laxatives recommended in traditional Persian medicine (PM) for constipation management (6). In the PM references, it is emphasized that this plant has more safety and fewer complications comparing with other laxative herbs, so it has been recommended as a useful drug in pregnant women and children (7-9). 

Constipation is one of the frequent complaints in pregnancy (10), and some herbal stimulant laxatives in *Cassia* genus seem to be safe during pregnancy; e.g studies have not shown the negative effects of senna (*Cassia senna*) during pregnancy (11). Nevertheless, considering its probable mutagenicity and genotoxicity (12), its use still remains controversial (13). Similar to senna, *C. fistula* could be effective in constipation as an anthraquinone laxative agent(14). The glycoside forms of anthranoid laxatives are non-absorbable in the small intestine. The large intestinal bacterial flora transforms the anthranoid into its pharmacologically active aglycon anthrone (15). Although the short-term oral use of anthranoids is generally considered safe, some studies demonstrated that they could be tumorigenic after a long-term exposure to high dosages and cause endometrial stimulation (16). To the best of our knowledge, no study has been carried out on the effect of *C. fistula* in maternal reproductive outcome and fetal anomaly incidence in rats. As *C. fistula *is widely used for the treatment of constipation of pregnant women in PM, and there has been no study found regarding its possible side effects in pregnancy, this study aimed to examine the effect of *C. fistula* on the incidence of abortion and congenital anomalies, as well as its effect on some hepatic, renal and electrolyte indices in pregnant rats.

## Methods

This study was carried out in the school of traditional Persian Medicine at Babol University of Medical Sciences, Iran. This study was approved by the Ethics Committee of Babol University of Medical Sciences (ethics committee co: Mubabol.REC.1394.112).

**Plant material preparation:** *C. fistula* dry fruits were purchased from herbal store in Tehran. The plant was identified and authenticated by a botanist. A voucher specimen (number 1176) has been deposited at Herbarium of Faculty of Pharmacy, Shahid Beheshti University of Medical Sciences, Iran. The pulp of the plant was separated and soaked in water at room temperature. After filtration, the obtained extract was evaporated to dryness at 40 °C by rotary evaporation, then the dry extract was stored at –5°C until further use. 

The obtained extract was dissolved in water via tween80 (10% v/v) and was administered orally at certain concentrations by gavage to the animals of each group.

**Anthraquinone** **‎** **determination via High performance liquid chromatography (HPLC) method: **Anthraquinone standard (Sigma) was prepared in different concentrations (12.5, 25, 50 and 100µg/ml). Extract sample and reference standard were analyzed using HPLC Kanuer (Germany) system with the following specifications: C18 column with 4.16 mm diameter & 250 mm length, UV detector wavelength of 250nm, mobile phase acetonitrile and water with 40/60 ratio and flow rate 0.4 ml/min. Anthraquinone was identified according to retention times as a comparison with the standard. The concentration was measured from the peak area according to calibration curve of standard. The amount was expressed as the percentage of the dry extract (% w/w) (14).

**Experimental animals: **Female Wistar rats weighing 180-220 g (n=72), were obtained from the animal house of Babol University of Medical Sciences (Babol, Iran). These animals were adapted and housed under controlled standard laboratory conditions (22±30C, 12-hr light/dark cycle), and free access to standard food and tap water ad libitum. The animals were cared in accordance with the guidelines for the Care and Use of Lab Animals.

**Acute toxicity test: **Extract of *C.fistula *firstly at the dose of 2500 and then at 5000mg/kg body weight were sequentially administered orally in 2 phases in the two groups in which each group contains six mice. Animals were observed for 48h after the extract administration for mortality and clinical signs of toxicity (restlessness, dullness, agitation) and followed-up for one week.

**Experimental design: **Female rats were divided into six experimental groups (n=12 animals/group) as follows: Group A: 500mg/kg, Group B: 1000mg/kg, Group C: 2000mg/kg of the extract was administered orally to the animals. Tween80 10% (100mg/kg) and distilled water were administered orally to the animals respectively in Group D and Group E. Group F was considered as the normal group. Animal weights were determined at the first of the study. Animals were administered extracts once a day (Q.D.) for three days. Then, all female rats were numbered with permanent marker in each group and housed (3 females and 1 male in per cage) males overnight and the appearance of a vaginal plug in the morning indicated that copulation and potential pregnancy occurred. Daily administering of the extract was continued in rats until the time of delivery. Maternal blood was collected from orbital sinus both 24h before intervention (for all rats) and after delivery (for pregnant rats). Samples were collected then centrifuged; and separated serums stored at -20 freezers until the time of evaluation of serum indices including Aspartate Aminotransferase (AST), Alanine Aminotransferase (ALT), urea, and creatinine (Cr) as well as serum potassium (K). During pregnancy, the rats were observed in term of mortality and abortion. After birth, newborn rats were counted and the number of stillbirths was recorded.

**Determination of serum indices: **Serum indices, Aspartate Aminotransferase (AST) and Alanine Aminotransferase (ALT) were determined via using kits of Biochemistry Company. Urea and Creatinine (Cr) were determined using the kits from Pars Azmoon Company and with the Biochemistry Auto-analyzer (Alpha Classic). The accuracy of the tests was also finally examined according to the standards (Tru lab p, n) and kits. Also, potassium (K) determination was carried out via the Ion selective electrode (ISE) method.

**Neonatal anomalies, histopathologic and** **histomorphometric evaluations: **All newborn rats were observed in term of weight and height after anesthesia; their crown–rump lengths (CRL) were measured. Then the selected slices of newborn rats fixed in 10% formalin, were processed using a tissue processor device (dehydration, clearing, and imbedded in paraffin); the blocks were cut at 5-micron thick serial section and then stained by the Hematoxylin and Eosin (H & E). Histological changes of brain, lung, heart, liver and kidney were assessed qualitatively under light microscope (Olympus). In histomorphometric study, malpighian body area and bowman’s capsule space were measured in kidney tissue using Motic Image Plus 2 software. On average, 9 slices were obtained from each rat neonate; and from each slice on average, 4 images were taken from different areas of the cortex at magnification of 40 x 40 and approximately one to three glomeruli in each image (300 glomeruli in each group) were measured.

**Statistical analysis: **The data were analyzed using Statistical Package for the Social Sciences (SPSS) 22. To describe the characteristics of the research units, descriptive statistics were used as mean (± standard deviation) and frequency (percentage). For comparison of the number of newborn rats and also the number of impaired tissues, chi-square was used. Also, for comparing the height and weight of newborn rats Analysis of variance (ANOVA) with Tukey’s multiple comparisons was used. In order to assess the changes in serum indices before and after intervention in each group, we used paired t-test. To examine the differences in the four groups of *C. Fistula* extracts at baseline, one way ANOVA was used. The difference of each serum level between before and after intervention in these four groups was examined by generalized estimating equation (GEE). In order to compare the means in different times or groups, we used pairwise contrasts at EM means in GEE. Differences were considered statistically significant when p<0.05.

To quantify the qualitative variables, we scaled non, mild, moderate, and severe with the scores 0, 1, 2, and 3, respectively. Then, to compare them, we used ANOVA and Tukey post hoc multiple comparisons. Furthermore, to compare each group with the control group, U Mann-Whitney was used. 

## Results

**Determination of anthraquinone** **‎** **content in** ***C. fistula*** **extract: **Because of the importance of anthraquinones in various *Cassia* species as the main phytochemicals relevant to their laxative property, determination of anthraquinone was performed in the present study to ensure the presence of active component in the fruit extract. According to HPLC analysis, the content of anthraquinone in the plant extract was 0.34% w/w of the dry extract. Figure 1 depicts the chromatograms of the anthraquinone standard (A) and identification of anthraquinone in *C. fistula* extract (B).

**Figure 1:**
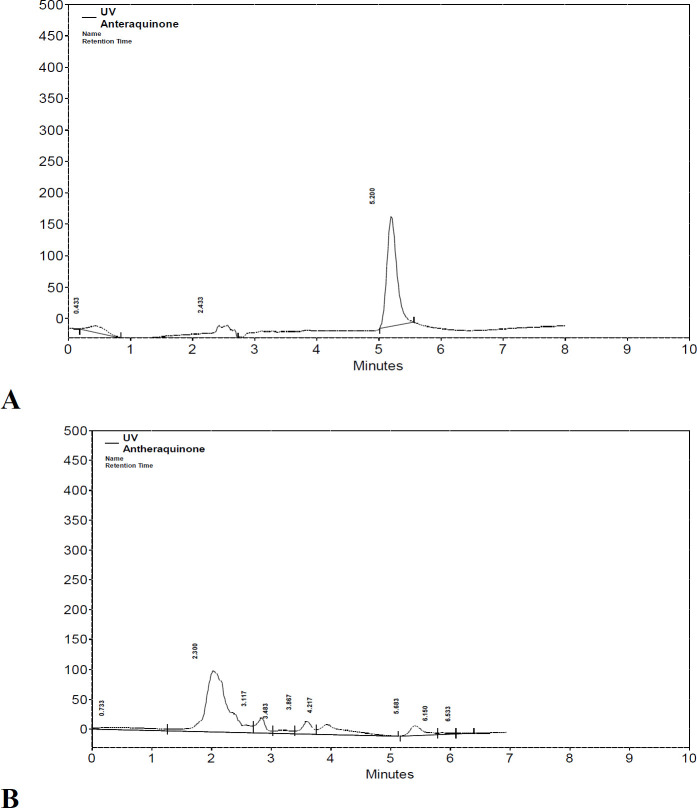
HPLC-chromatogram of anthraquinone standard (A). HPLC-chromatogram of anthraquinone determination in aqueous extract of C. fistula (B)

**LD50 determination: **After the gavage of the dose of 2500mg/kg and follow-up of the condition of the mice, no death occurred. Subsequently, in the second phase, six adult male mice were examined by a dose of 5000 mg/kg. Follow-up of mice was done according to the previous procedure and there was no death in the intended period. Due to no observation of mortality in the tested doses, LD50 was practically estimated more than 5000 mg/kg (LD50>5000 mg/kg).

**Reproductive outcomes and offspring characteristics: **The pregnancy rate and number of offspring were not significantly different between groups. The height and weight of the offspring, in groups A, C and D were reduced significantly compared to the E and F groups (p<0.001). The height and weight of group B (p=0.008) was higher among the groups receiving the extract, without any significant difference with groups E and F. The details have been shown in table 1. During the study, no abortions were observed in any of the groups. No anomalies were seen in any of the newborns.

**Table 1:** Rate of pregnancy and data of offspring in the studied group

**Groups (n=12)**	**A (500mg/kg)**	**B** **(1000mg/kg)**	**C (2000mg/kg)**	**D** **(tween80)**	**E** **(water)**	**F (normal)**	**P-value**
Pregnancy (Frequency (%))	6(50%)	5(41.6%)	6(50%)	6(50%)	6(50%)	6(50%)	0.989
Number of offspring in each pregnancy (mean±SD)	4.25±0.50	3.3±0.58	4±1.08	3±1.41	3±0.82	3.5±0.58	0.362
Height of offspring (mm) (mean±SD)	48.18±3.81^a^	52.70±0.95^b^	49.50±1.8^a^	49.42±1.8^a^	55.25±3.86^b^	55.86±4.54^b^	0.001>
Weight of offspring (g) (mean±SD)	6.52±1.62^a^	8.22±0.50^b^	6.78±0.74^a^	7.46±0.56^a^	9.15±1.73^b^	9.06±2.05^b^	0.001>

A; 500mg/kg; B; 1000mg/kg; C; 2000mg/kg; D; Tween80, 10%, E: Water, F: Normal groups with no statistical significant difference via chi-square test (α=0.05). a and b: non-uniform letters in each level indicate a significant difference at α = 0.05.

**Serum indices: **Blood urea in groups B, C, and D, after intervention decreased, but it was only significant in group B (P=0.02). The changes of Cr and K in each group were not significant comparing before and after the intervention. The AST level decreased significantly after intervention in groups A, B and C. The level of ALT increased significantly only in group C but remained unchanged in other groups comparing before and after the intervention. The details are shown in table 2.

**Table 2:** Comparison of serum parameter before and after intervention *(C. fistula extract)* in pregnant rats

**Parameter (mean±SD)**	**Intervention**	**A** **500mg / kg**	**B** **1000mg / kg**	**C** **2000mg / kg**	**D** **Tween80,10%**	**P-value** ^*^
Blood urea	Before	45.69±5.06	49.44±3.84	46.93±3.49	44.56±5.95	0.091
	after	46.06±5.10	42.94±6.42^**^	43.38±4.44	43.31±3.70	
Creatinine	Before	0.74±0.07	0.76±0.06	0.71±0.09	0.73±0.08	0.984
	after	0.72±0.05	0.75±0.05	0.69±0.05	0.72±0.05	
K (Potassium)	Before	6.07±0.55	6.02±0.44	5.84±0.40	6.04±0.54	0.871
	after	6.14±0.37	6.09±0.21	6.02±0.31	6.29±0.26	
AST ()	Before	177.37±36.21	174.75±30.12	164.21±40.73	128.50±22.95	0.016
	after	124.88±34.98^**^	122.50±27.63^**^	117.25±20.29^**^	143.94±54.27	
ALT ()	Before	53.19±8.34	60.13±15.56	51.00±8.66	61.44±9.85	0.366
	after	65.56±18.08	68.71±15.23	63.43±10.49^**^	61.38±12.53	

AST (Aspartate Aminotransferase), ALT (Alanine Aminotransferase)

*P-value for interaction of time and groups ** Significant difference in each group before and after intervention with EM means test in GEE at the level of α = 0.05.

**Histopathologic and histomorphometric evaluations: **No significant pathological findings were observed in brain tissues compared to control group. One case of necrosis was seen in the different brain regions of group D, but was not statistically significant. Pathologic findings in terms of inflammation, collapse, emphysema, and necrosis were not observed in lung tissues in any of the groups. Alveolar secretions (exudates) were observed in only one case in group B and one in group C, which were not statistically significant. Pulmonary vascular congestion was observed in the right lung of all groups but this finding was not significant between the groups. Left lung vascular congestion was observed in all groups it was only significant in group D compared to control group (P=0.04) (Figure 1). Hypertrophy in heart tissue was observed in groups D and B which was significant only in group D (P=0.01) (Figure 2). Congestion of cardiac cells was also seen in these two groups but was not statistically significant. Among the different parameters studied in liver tissue, only parenchymal inflammation in groups D (P=0.004) and C (P= 0.026) and B (P=0.042) was statistically significant (Figure 3). Necrosis was observed only in one case in each group of B and C which was not statistically significant. Hepatocytes vacuolation and destruction were observed in all groups with no significant difference with control group.

In all groups, white and red pulp structure in the spleen tissue was similar to the control group. No significant finding was found in any of the indices assessed in kidney tissues. There was just one case of extensive necrosis of renal tissue in group D which was not statistically significant (Figure 4). Glomerular malformations were observed in one case of group B and one case of group D, which were not statistically significant. 

In case of histomorphometric parameters, in all tissues of all groups, malpighian body area and bowman’s capsule space significantly increased compared to the control group (table 3).

**Table 3:** Comparison of malpighian corpuscle area and bowman’s capsule space in the study groups

**Groups ** **Offspring **	**A** **500mg / kg**	**B** **1000mg / kg**	**C** **2000mg / kg**	**D** **Tween80,10%**	**E** **(water)**	**P-value**
Malpighian corpuscle area (µm)2	28323.05 ±11677.65*	27676.80 ±11084.31*	30979.78 ±12183.32*	33309.36 ±15642.52*	21356.55 ±7022.94	<0.001
Malpighian corpuscle area (µm)2	11921.29±5387.45*	14818.02±5754.13*	13259.82±7490.58*	13750.30±7030.24*	5753.10±3029.83	<0.001

A: 500 mg /kg, B: 1000 mg / kg, C: 2000 mg / kg, D: tween80.10%, E: water. The numbers in the table: Mean ± SD of the cross-sectional area of the malpighian body area and bowman’s capsule space. *: Significant difference at α = 0.001 level with control group

**Figure 1:**
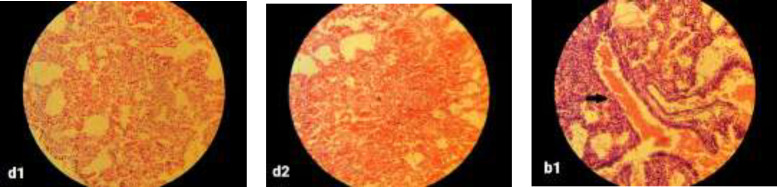
Histological image of the lung: d1 and d2 show low- and high-intensity pulmonary hemorrhage in group D, respectively, and b1 shows high-intensity pulmonary hemorrhage in group B, including bronchial blood.   40 × magnification, H&E

**Figure 2:**
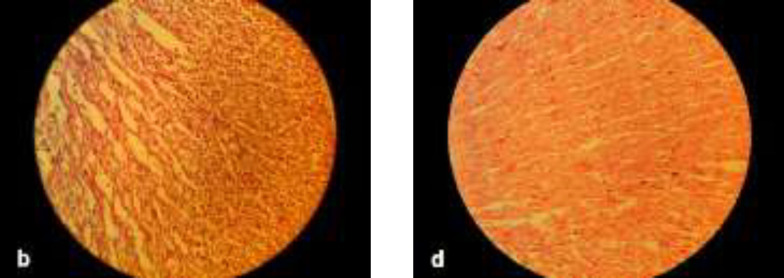
Histological images of the heart. Hypertrophy is seen in groups B (image b) and D (image d). 40 × magnification

**Figure 3:**
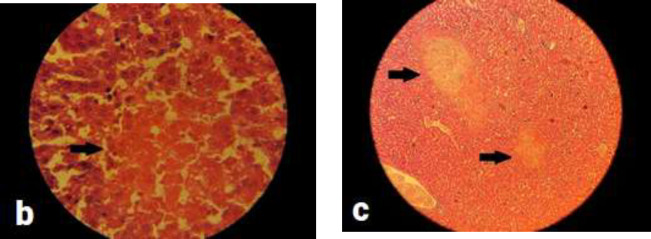
Histological images of the liver in groups B (image b) and C (image c). Images show necrosis in the liver

**Figure 4:**
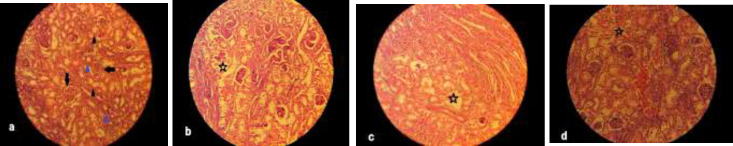
Histological images of the kidney in groups A, B, C and D (images a, b, c, and d, respectively). Black arrows: Malphigian body, Black triangles: Proximal tubule, Blue triangle: Distal tubule, Stars: Tubular destruction areas.40 × magnification, H&E

## Discussion

In the present study, *C. fistula *aqueous extract was shown as an agent with a low adverse effect in pregnant rats. *C. fistula* is generally used as a medication with low side effect for the treatment of diseases such as constipation. In traditional Persian medicine books, with emphasis on its low adverse effects and lack of complication, it is recommended to treat pregnant women and children with constipation (7). The amount of LD50 of *C. fistula *aqueous extract (> 5000mg/kg) in the present study is consistent with the study of Akanmu et al. (17), which showed that oral administration of the *C. fistula* infusion for six weeks at doses 250, 500 and 1000 mg/kg was found not to produce any changes in rats’ behavior and health items and no macroscopic alterations were found in their liver, kidney, testis and brain. It can be suggested that the drug is non-toxic or very low toxic, in other words, it might be considered as a safe drug. Moreover, Jothy et al. (18), showed that *C. fistula* seeds’ methanolic extract, even at a dose of 5,000 mg/kg did not have any adverse effect on serum and macroscopic indices in mice, and confirmed the safety of this herbal medicine. 

Generally, in the present study *C. fistula* extract did not significantly affect pregnant rats. However, in offspring of rats, some changes have been observed. But these alterations can be attributed to the effect of the tween 80. The tween reduced the height and weight in the offspring of the rats. The observation of its complications reinforces the notion that tween cannot be harmless. Moreover, it might be notable that as a secondary outcome, the medium-dose (1000mg/kg) of *C. fistula *extract seems to be optimum and to some extent could reduce the potential side effects of the tween 80. There was no abortion in any of the groups during the pregnancy. The fact that *C. fistula* has not led to any abortion is one of the important achievements of this study. Also, the number of newborns in the pregnant groups did not differ significantly. No anomalies were seen in the offspring of any groups of pregnant rats receiving *C. fistula*.

In the analysis of the biochemical findings, there was no significant difference in urea level between groups while *C. fistula *extract showed significant decrease in urea level after intervention only in the group B (1000mg/kg). In case of creatinine, there was not any significant alteration observed in creatinine level in intervention and control groups. In Teresa's study (19) there was also no significant reduction in the amount of creatinine after a physiological pregnancy, as well as in the study by Urasoko (20). In the study of de Rijk et al. (21), a significant reduction in creatinine after pregnancy was reported. The range of creatinine reported in our study is very close to the study of Urasoko (20) and Gross (22) but higher than that in Teresa *et al*.’s (19) study. Teresa (19) and Urasoko (20) reported an increase in potassium after physiologic pregnancy. While no significant changes in potassium were observed in any groups of our study (23). Two most reliable markers of hepatocellular damage, AST and ALT, increased in various hepatic dysfunctions. Of these two enzymes, ALT is more specific for liver damage because it is mainly found in the cytosol of the liver and in low concentrations elsewhere (24). 

The elevated ALT in serum of the animals in group C shows that the higher concentration of *C. fistula *extract (2000 mg/kg) might result in hepatocellular damage. In case of pregnancy itself, Urasoko et al.,(20), reported that ALT increased significantly after pregnancy in rats, while in Teresa’s(19) and EVELINE ‘studies (21) the ALT had an insignificant increase; In our study in pregnant rats, there was no significant change in ALT, after intervention in control group. 

In the study of Teresa *et al.*,(19), AST after pregnancy had a mild increase (3%); while in the study of Urasoko (20), AST remained statistically unchanged after pregnancy. In the present study, we observed a significant dose-dependent reduction in AST after administration of *C. fistula* extract in groups A, B and C compared to control group. However, there was not any significant change in AST level after pregnancy in control group. The range of AST reported in our study before pregnancy was similar to that of Gross (22) and more than that in Teresa’s study (19). It seems that pregnancy had no effect on urea, potassium, Cr, ALT and ALS levels in the comparisons between before and after intervention in control group. Some studies have been performed on histopathologic evaluation of rats receiving different parts of *C. fistula* (pods, seed, fruit, etc.) (18, 25). 

But no study has reported the effects of *C. fistula* on neonates of rats receiving *C. fistula*. In the present study, histopathological examination of brain, spleen and kidney tissues showed no statistically significant findings. Only in the group that received tween80, we observed statistically significant left lung congestion, hypertrophic changes in heart tissue, and mononuclear cell aggregation in the liver tissue parenchyma. Also, in quantitative evaluation of malpighian corpuscle cross-sectional area and bowman capsule space, we observed a statistically significant increase in all groups compared to the control group with the most different changes in group D (tween 80). In animal studies on polysorbate 80 (oral route), there were no reports on its effects on fertility, morphological development, survival and growth of fetuses. Tween80 was not reported as a teratogenic agent (26). Our initial assumption in this study was that tween80 had no specific side effects; however, it seems that tween80 alone caused some pathological changes in the neonates. Concerning the confounding role of tween, the effect of *C. fistula* on histopathological alterations needs to be more studied. On the other hand, considering the side effects of tween80 in this study, although *C. fistula* with a dose of 1000 mg/kg reduced some of the embryonic effects of tween80, a final decision on the possible complications of fetus in pregnancy requires further studies. The confounding effect of tween80 and not including its different concentrations could be considered as the weakness of our study.

As *C. fistula* is widely used for treatment of constipation of pregnant women in PM, and there has been no study found regarding its possible side effects in pregnancy, investigating the effects of *C. fistula* in maternal reproductive outcome and fetal anomaly incidence in rats for the first time can be the strength of the present study. 

In conclusion *C. fistula* with an LD50 of more than 5000 mg/kg orally in female rats can be considered as a low-toxic and safe drug. There was not any significant change in the pregnancy rate, and number of offspring. About the decrease in height and weight of the offspring, and renal histomorphometric changes, it cannot be concluded as direct effect of the *C. fistula* administration, because of the confounding role of tween80. In biochemical analysis, it can be concluded that the higher concentration of *C. fistula *extract (2000 mg/kg) might lead to ALT elevation showing hepatocellular damage. It seems that pregnancy itself had no effect on urea, potassium, and Cr, ALT and ALS levels as seen in the comparisons between before and after intervention in control group.

The use of *C. fistula* as the drug for constipation in pregnancy requires more clinical evidence, and the data from this study cannot be enough for long-term treatment with this herbal medicine. However, given that the dose of 1000 mg/kg of *C. fistula* has been associated with protective effects on offspring’s height and weight changes, it is recommended that this dose might be used in the future clinical studies. Despite of no abortions and anomalies of offspring of pregnant rats receiving *C. fistula *in this study, more research on microscopic changes in the neonates are also necessary to conclude that there is a low incidence of complications during pregnancy.
